# Alcohol Consumption within 48 hours before Onset Is Associated with Adverse Clinical Outcomes in Hypertriglyceridemic Pancreatitis

**DOI:** 10.3390/jcm12072566

**Published:** 2023-03-29

**Authors:** Tianming Lai, Yin Zhu, Nonghua Lu, Wenhua He

**Affiliations:** Department of Gastroenterology, Digestive Disease Hospital, The First Affiliated Hospital of Nanchang University, 17 Yong Wai Zheng Street, Nanchang 330006, China; laitianming1997@126.com (T.L.);

**Keywords:** alcohol consumption, hypertriglyceridemic pancreatitis, severity, outcomes

## Abstract

(1) Background: Some patients with hypertriglyceridemic pancreatitis (HTGP) drink occasionally or moderately, but do not meet the diagnostic criteria for alcoholic pancreatitis. This study aims to investigate whether occasional or moderate alcohol consumption affects the clinical outcomes of patients with HTGP. (2) Methods: This retrospective study included 373 patients with HTGP from January 2007 to December 2021. HTGP patients with occasional or moderate alcohol (OMA) consumption before onset were divided into the OMA group, and HTGP patients without alcohol (WA) consumption were divided into the WA group. The OMA group was further divided into two groups: the drinking within 48 h before onset (DW) group, and the without drinking within 48 h before onset (WDW) group. The clinical data of the two groups were compared and multivariable logistic regression was used to analyze independent risk factors for the primary outcomes. (3) Results: The proportion of men (95.7% vs. 67.6%, *p* < 0.001) and smoking history (61.7% vs. 15.1%, *p* < 0.001) in the OMA group were higher than those in the WA group. Occasional or moderate alcohol consumption was independently associated with a high incidence of SAP (adjusted odds ratio (AdjOR), 1.57; 95% CI, 1.02–2.41; *p* = 0.041), and necrotizing pancreatitis (AdjOR, 1.60; 95% CI, 1.04–2.48; *p* = 0.034). After dividing the OMA group into two subgroups, we found that drinking within 48 h before onset was independently associated with a high incidence of SAP (AdjOR, 3.09; 95% CI, 1.66–5.77; *p* < 0.001), and necrotizing pancreatitis (AdjOR, 2.71; 95% CI, 1.46–5.05; *p* = 0.002). (4) Conclusion: Occasional or moderate alcohol consumption is associated with poor clinical outcomes in patients with HTGP, particularly if they drank alcohol within 48 h before the onset of the disease.

## 1. Introduction

Acute pancreatitis (AP) is an inflammatory state of the pancreas, which can lead to local injury, systemic inflammatory syndrome, and organ failure [1]. It is the most common gastrointestinal disease requiring emergency hospitalization [2]. Worldwide, the incidence of AP is on the rise [3]. Among the many causes of AP, pancreatitis caused by hypertriglyceridemia (HTG) is more severe and has a worse prognosis than AP caused by other causes [4,5,6]. Hypertriglyceridemic pancreatitis (HTGP) has shown a rising trend in recent years, and has leaped to become the second leading cause of AP in China [6,7,8,9].

According to the definition of the etiology of AP in the American College of Gastroenterology guidelines, alcohol can be considered to be the cause of AP if a patient has a history of over 5 years of heavy alcohol consumption (>50 g/per day) [10]. Therefore, alcohol as an etiology of AP usually occurs in patients with a long history (>5 years) of heavy drinking (>50 g/d). In other cases (occasional or moderate alcohol consumption, where the diagnostic criteria for alcoholic pancreatitis are not met due to the patient’s low alcohol consumption or the patient’s short history of alcohol consumption), alcohol cannot be considered as an etiology of AP. However, it is unclear whether occasional or moderate alcohol consumption affects the clinical outcomes of patients with HTGP.

A previous study [11] showed that episodic heavy alcoholic consumption before the onset of symptoms could aggravate the outcomes of first-episode severe acute pancreatitis (SAP) and increase the incidence of organ failure, local complications, and mortality in these patients. However, the baseline information of this study (such as etiology, gender, etc.) was not balanced between the groups, resulting in confounding bias to some extent, and the study did not consider the patient’s history of alcohol consumption. Until now, few studies have investigated whether occasional or moderate alcohol consumption affects the clinical outcomes of patients with HTGP. Therefore, elucidating their relationship can help to improve prognosis and quality of life through timely clinical intervention in such patients. In this study, we explored whether occasional or moderate alcohol consumption may worsen the clinical prognosis of patients with HTGP.

## 2. Materials and Methods

### 2.1. Study Design and Participants

**Figure 1:**
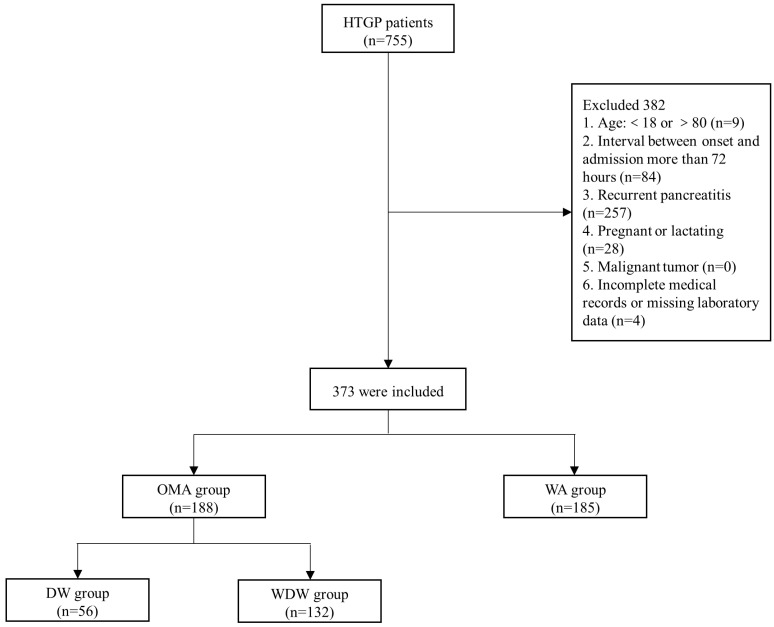
Flow of chart.

This was a retrospective cohort study, analyzing patients with first episodes of HTGP who were hospitalized in the Department of Gastroenterology of the First Affiliated Hospital of Nanchang University from January 2007 to December 2021. All data were obtained from the AP database of the First Affiliated Hospital of Nanchang University. The AP database was a prospectively maintained database that collected the data of AP inpatients, including basic information, diagnosis, treatment, etc. The study was approved by the Ethics Committee of the First Affiliated Hospital of Nanchang University (No. 2011001). In this study, the exclusion criteria were as follows: (1) age < 18 years old or >80 years old; (2) interval between onset and admission (more than 72 h); (3) recurrent pancreatitis; (4) pregnant or lactating; (5) malignant tumor; (6) incomplete medical records or lack of laboratory data. After excluding patients who did not meet the criteria, we divided the patients into two groups according to whether they had consumed alcohol before the onset of AP: the occasional or moderate alcohol (OMA) group and the without alcohol (WA) group. The OMA group was further divided into two groups, according to whether they had consumed alcohol within the 48 h before the onset of AP: the drinking within 48 h before onset (DW) group, and the without drinking within 48 h before onset (WDW) group. The specific inclusion process was shown in Figure 1. Finally, we collected the data on gender, age, body mass index (BMI), history of smoking, referral or non-referral, comorbidities, laboratory tests, acute physiology and chronic health evaluation (APACHE II) score, Ranson score, CT severity index (CTSI) score, severity, complications, and prognosis of the included patients. Among these, baseline information, laboratory tests, the APACHE II score, and the CTSI score were evaluated within 24 h of admission, and the Ranson score was evaluated within 48 h of admission. Some studies showed that Ranson ≥ 3 and APACHE II ≥ 8 were good predictors of poor prognosis in acute pancreatitis [12,13]. Therefore, we counted the number of people with Ranson ≥ 3, APACHE II ≥ 8, and CTSI ≥ 4 in each group.

### 2.2. Definition

In this study, the diagnostic criteria of HTGP was defined as an increase in serum triglyceride (TG) greater than 1000 mg/dL in the absence of gallstones, and/or a significant history of alcohol use [10]. The levels of TG were measured at the time of admission and were mixed admission samples (they were not all fasting samples). Occasional or moderate alcohol consumption was defined as when the patients consumed alcohol before the onset of the disease but did not meet the diagnostic criteria of alcoholic AP, which was defined as a history of over 5 years of heavy alcohol consumption (>50 g/per day) [10]. In other words, alcohol intake was below the threshold of alcoholic AP in all patients in the OMA group. These patients included those who had alcohol intake immediately prior to the onset of AP, or who had simply consumed alcohol at some point prior to AP onset. All patients in the WA group had no history of alcohol consumption. The primary outcomes included SAP and necrotizing pancreatitis. SAP was defined by the presence of organ failure for more than 48 h [14]. Necrotizing pancreatitis was confirmed by a nonenhanced area of the pancreatic parenchyma, peripancreatic tissue, or both on contrast-enhanced CT [14]. Specific definitions of the primary and secondary outcomes are shown in Table S1.

### 2.3. Study Outcomes

In this study, the primary outcomes were the proportions of SAP and necrotizing pancreatitis. The secondary outcomes included persistent organ failure, persistent multiple organ failure (MOF), abdominal compartment syndrome (ACS), sepsis, infected pancreatic necrosis (IPN), organ function support, local complications, intervention of local complications, intensive care unit (ICU) admission, death, length of hospital stay, and total hospital costs.

### 2.4. Statistical Analysis

Data were analyzed using SPSS 25.0 software (Chicago, IL, USA) or R software (version 4.1.0, R Development Core Team). Continuous variables were presented as the medians (interquartile ranges, IQR) or the means ± standard deviations, and were analyzed using the Mann–Whitney U test or t-test, as appropriate. Categorical variables were presented as absolute numbers and proportions. The Chi-square test or Fisher’s exact test was used for pairwise comparison of proportions. Factors associated with primary outcomes in unadjusted models (*p* < 0.2) were included in the multivariable models to identify the risk factors independently associated with primary outcomes. All results were presented as odds ratios (ORs) and 95% confidence intervals (CIs). A two-tailed *p* value < 0.05 was considered statistically significant.

## 3. Results

### 3.1. Baseline Characteristics, Clinical Scoring Systems and Laboratory Findings of Patients in the OMA Group and WA Group

A total of 755 patients diagnosed with HTGP were screened; of these patients, 373 patients who met the inclusion criteria were enrolled, with 188 patients in the OMA group and 185 patients in the WA group (Figure 1). Of these 373 patients, a total of 305 (81.8%) were males and 68 (18.2%) were females. Their median age was 41.0 (IQR: 14.0) years and their median BMI was 25.9 (IQR: 3.6). Baseline demographic characteristics, comorbid conditions, clinical scoring systems, and laboratory findings of the patients in the OMA group and WA group are shown in Table 1 and Table 2. The proportions of men and smoking history in the OMA group were higher than those in the WA group (95.7% vs. 67.6%, *p* < 0.001; 61.7% vs. 15.1%, *p* < 0.001). There were no statistical differences between the two groups in terms of age, BMI, transfer status, and comorbidities (all *p* > 0.05). As shown in Table 2, when focusing on the clinical scoring systems, the proportion of patients with APACHE II ≥ 8 was higher in the OMA group than in the WA group (48.4% vs. 34.1%, *p* = 0.005). Compared to patients in the WA group, patients in the OMA group had higher hematocrit (HCT) (44.9(IQR: 8.1) vs. 44.0(IQR: 7.6), *p* = 0.027), serum aspartate transaminase (AST) levels (33.0(IQR: 29.0) vs. 27.0(IQR: 22.0), *p* = 0.007), serum total bilirubin (TB) levels (15.2(IQR: 12.2) vs. 13.0(IQR: 9.9), *p* = 0.015), serum direct bilirubin (DB) levels (3.8 (IQR: 3.4) vs. 3.0(IQR: 2.8), *p* = 0.002) and serum creatinine (Cr) levels (68.0(IQR: 45.9) vs. 62.7(IQR: 35.4), *p* = 0.033).

### 3.2. Clinical Outcomes of Patients in the OMA Group and WA Group

As shown in Table 3, the proportion of SAP was 41.0% in the OMA group and 30.8% in the WA group (*p* = 0.041). The proportion of necrotizing pancreatitis was 40.4% in the OMA group and 29.7% in the WA group (*p* = 0.030). Moreover, patients in the OMA group had a significantly higher proportion of persistent organ failure (41.0% vs. 30.8%, *p* = 0.041), persistent respiratory failure (40.4% vs. 29.2%, *p* = 0.023), persistent MOF (18.1% vs. 9.2%, *p* = 0.012), mechanical ventilation (25.5% vs. 14.6%, *p* = 0.008), and ANC (40.4% vs. 29.7%, *p* = 0.030) than patients in the WA group, and they also had higher hospital costs (31118(50560) vs. 23999(33651), *p* = 0.016).

### 3.3. Multivariate Logistic Regression Analysis for Primary Outcomes in OMA Group and WA Group

The univariate results of patients with SAP and necrotizing pancreatitis are detailed in Tables S2 and S3, respectively. Factors associated with adverse clinical outcomes in unadjusted models (*p* < 0.2) were included in the multivariable models. As shown in Table 4, multivariate analysis showed that occasional or moderate alcohol consumption was independently associated with a high incidence of SAP (adjusted odds ratio (AdjOR), 1.57; 95% CI, 1.02–2.41; *p* = 0.041), and necrotizing pancreatitis (AdjOR, 1.60; 95% CI, 1.04–2.48; *p* = 0.034).

### 3.4. Subgroup Analysis

#### Baseline Characteristics, Clinical Scoring Systems, Laboratory Findings and Clinical Outcomes of the Patients in the DW Group and the WDW Group Were Analyzed

To further explore the relationship between alcohol consumption and the prognosis of HTGP, a subgroup analysis was conducted. Patients in the OMA group were divided into the DW group (n = 56) and the WDW group (n = 132) according to whether they had consumed alcohol within 48 h prior to the onset of AP. As shown in Tables S4 and S5, the baseline characteristics were equally distributed between the DW group and the WDW group. There were no significant differences in clinical characteristics, including sex, age, BMI, transfer status, history of smoking, and comorbidities between the two groups (all *p* > 0.05). Compared to patients in the WDW group, patients in the DW group had higher AST levels (40.0(IQR: 45.0) vs. 30.0(IQR: 24.0), *p* = 0.045) and serum TB levels (16.0 (IQR: 12.9) vs. 14.0(IQR: 12.6), *p* = 0.048). As shown in Table 5, for primary outcomes, patients in the DW group had a higher proportion of SAP (57.1% vs. 34.1%, *p* = 0.003) and necrotizing pancreatitis (53.6% vs. 34.8%, *p* < 0.017) than patients in the WDW group. As for secondary outcomes, patients in the DW group had higher persistent organ failure (57.1% vs. 34.1%, *p* = 0.003), persistent respiratory failure (55.4% vs. 34.1%, *p* = 0.007), ANC (53.6% vs. 34.8%, *p*< 0.017), and ICU admission (57.1% vs. 34.8%, *p* = 0.005). They also had a longer stay in hospital (16.5 (IQR: 16.0) vs. 11.0 (IQR: 9.0), *p* = 0.019) and higher hospital costs (51366(89833) vs. 26603(38711), *p* = 0.008).

### 3.5. Multivariate Logistic Regression Analysis for Primary Outcomes among DW Group, WDW Group and WA Group

To further investigate which subgroup in the OMA group had a greater effect on the prognosis of HTGP, we again performed univariate and multivariate logistic regression analyses. The results of the univariate of patients’ demographic characteristics for SAP and necrotizing pancreatitis are detailed in Tables S6 and S7, respectively. Factors associated with adverse clinical outcomes in unadjusted models (*p* < 0.2) were included in the multivariable models. As shown in Table 6, multivariate analysis showed that drinking within 48 h before onset was independently associated with a high incidence of SAP (AdjOR, 3.09; 95% CI, 1.66–5.77; *p* < 0.001) and necrotizing pancreatitis (AdjOR, 2.71; 95% CI, 1.46–5.05; *p* = 0.002).

## 4. Discussion

In this study, we found that the proportion of the APACHE II score ≥ 8, HCT levels, serum AST levels, serum bilirubin levels and serum Cr levels within 24 h of admission were higher in the OMA group than in the WA group. Occasional or moderate alcohol consumption was independently associated with poor outcomes in patients with first-episode HTGP, including increasing the incidence of SAP and necrotizing pancreatitis. Subgroup analysis showed that patients in the DW group had higher AST levels, serum TB levels, a higher proportion of SAP, necrotizing pancreatitis, persistent organ failure, ANC, and ICU admission than those in the WDW group. Multivariate logistic regression analysis of the three groups showed that drinking within 48 h before onset was independently associated with a high incidence of SAP and necrotizing pancreatitis.

Our study found that patients in the OMA group had higher serum AST levels and TB levels than those in the WA group. In a subgroup analysis, patients in the DW group also had higher serum AST levels and TB levels than those in the WDW group. This may be associated with abnormal liver function due to alcohol consumption, especially patients drinking within 48 h before the onset of AP. In addition, the proportion of APACHE II ≥ 8, HCT, and serum Cr levels within 24 h of admission were higher in the OMA group than in the WA group, and the elevation of these indexes could predict the poor prognoses of AP. This indirectly indicated, to some extent, that occasional or moderate alcohol consumption was associated with poor prognoses in patients with first-episode HTGP. Although we found a correlation between occasional or moderate alcohol consumption and poor outcomes of HTGP, how occasional or moderate alcohol consumption altered or exacerbated disease progression in these patients was not well understood.

In our study, we found that the proportion of persistent respiratory failure was significantly higher in the OMA group than in the WA group, and in a subgroup analysis, patients in the DW group also had a higher proportion of persistent organ failure than those in the WDW group. This was similar to the findings of Deng et al. who found that pre-onset binge drinking increased the incidence of respiratory failure in patients with severe acute pancreatitis [11]. We speculated that it may be related to lung injury caused by alcohol consumption. It has been shown that alcohol could induce oxidative stress and dysfunction of alveolar macrophages, thus causing lung injury [15]. However, the underlying mechanism of why alcohol consumption within the 48 h prior to the onset of AP increased persistent respiratory failure in patients with HTGP remained unclear. In addition, patients in the OMA group had a higher proportion of necrotizing pancreatitis than those in the WA group, and in a subgroup analysis, patients in the DW group also had a higher proportion of necrotizing pancreatitis than those in the WDW group, which might be related to the pancreatic injury caused by alcohol metabolism. It has been shown that alcohol could promote the formation of fatty acid ethyl esters through the inhibition of oxidative pathways, thereby inducing a sustained elevation of cytosolic calcium, leading to the inhibition of mitochondrial function and necrosis of isolated pancreatic acinar cells [16]. The proportions of persistent respiratory failure and pancreatic necrosis were higher in the OMA group or in the DW group. In addition to the above possible mechanisms, we speculated that another possible mechanism was that by affecting TG metabolism, alcohol consequently led to elevated TG, which led to the above phenomena. A study found that alcohol may elevate TG by affecting TG metabolism [17]. Acute alcohol intake can reduce lipolysis of circulating chylomicrons and VLDL by reducing the activity of lipoprotein lipase [17,18]. When alcohol was consumed with a meal, the postprandial lipemic response was prolonged and raised fasting triglycerides the next morning [17,19]. The higher the TG level, the higher the incidence of persistent organ failure [20]. Another animal experiment found that the higher the TG level, the more severe the pancreatic injury and the higher the incidence of pancreatic necrosis [21]. However, in our study, although TG levels were higher in the OMA group than in the WA group, and higher in the DW group than in the WDW group, there was no statistical difference in TG levels between the groups (18.1(13.0) vs. 18.0(13.1), *p* = 0.621; 20.0(13.6) vs. 17.9(12.0), *p* = 0.584, respectively).

In conclusion, our study found that patients in the OMA group were more severe than those in the WA group, and patients in the DW group were more severe than those in the WDW group. These findings were mainly associated with drinking within 48 h before the onset of AP. This was consistent with the findings of a previous study, which found that pre-onset binge drinking exacerbated the outcomes of first-attack severe acute pancreatitis [11]. The hypothesis for the pathogenesis of HTGP was that pancreatic lipase enters the pancreatic vascular bed to decompose serum TG, producing high levels of free fatty acids (FFAs) and causing damage to pancreatic acinar cells and capillaries [22]. Oleic acid (OA) in FFAs plays a central role in causing extrapancreatic organ failure [23]. We hypothesized that drinking increased the production of pancreatic lipase by stimulating the secretion of pancreatic juice, leading to more leakage of pancreatic lipase into the vascular bed of the pancreas, which in turn aggravated the condition. Therefore, it is important to abstain from alcohol. In this study, we reported for the first time the relationship between occasional or moderate alcohol consumption and the clinical prognoses of first-episode HTGP. However, this study had some limitations. Firstly, this was a single-center study, which limited the representativeness of the study and therefore, it may not apply to other hospitals or communities; secondly, as a retrospective study, there was information bias; and finally, again since this was a retrospective study, we were unable to obtain the amount of alcohol consumption of some patients in the OMA group. Therefore, we were unable to calculate the mean alcohol intake of patients in the OMA, DW and WDW groups. In future, prospective studies will be needed to collect this data.

## 5. Conclusions

In conclusion, our study found that occasional or moderate alcohol consumption was associated with poor clinical outcomes in HTGP, especially for patients who drank alcohol within 48 h before the onset of AP. Thus, occasional or moderate alcohol consumption may be a contributor to the exacerbation of HTGP, especially if consumed within 48 h before the onset of AP. However, the exact mechanisms need to be further elucidated by additional studies.

## Figures and Tables

**Table 1:** Comparison of clinical characteristics in patients in OMA group and WA group.

Variables	Total	OMA Group (n = 188)	WA Group (n = 185)	*p*
Sex(male), n (%)	305 (81.8)	180 (95.7)	125 (67.6)	**<0.001**
Age, years, IQR	41.0 (33.0–47.0)	42.0 (35.0–47.0)	40.0 (31.0–46.0)	0.204
BMI, kg/m^2^, IQR	25.9 (24.0–27.6)	26.1 (24.2–27.8)	25.5 (23.9–27.4)	0.272
Transfer status, n (%)	218 (58.4)	110 (58.5)	108 (58.4)	0.979
History of smoking, n (%)	144 (38.6)	116 (61.7)	28 (15.1)	**<0.001**
Comorbidities, n (%)				
Hypertension	65 (17.4)	31 (16.5)	34 (18.4)	0.631
Diabetes mellitus	76 (20.4)	40 (21.3)	36 (19.5)	0.663
Hyperlipidemia	81 (21.7)	44 (23.4)	37 (20.0)	0.425
COPD	2 (0.5)	1 (0.5)	1 (0.5)	1
Coronary artery disease	1 (0.3)	1 (0.5)	0 (0)	1

OMA, occasional or moderate alcohol; WA, without alcohol; IQR, interquartile range; BMI, body mass index; COPD, chronic obstructive pulmonary disease.

**Table 2:** Comparison of clinical scoring systems and laboratory findings in patients in OMA group and WA group.

Variables	Total	OMA Group (n = 188)	WA Group (n = 185)	*p*
Clinical score systems, n (%)				
CTSI score ≥ 4	157 (42.1)	82 (43.6)	75 (40.5)	0.547
Ranson score ≥ 3	203 (54.4)	102 (54.3)	101 (54.6)	0.948
APACHE II score ≥ 8	154 (41.3)	91 (48.4)	63 (34.1)	**0.005**
Laboratory findings, median (IQR)				
WBC, × 10^9^/L	13.1 (10.4–16.1)	12.6 (10.0–16.0)	14.0 (11.1–17.2)	**0.013**
Hb, g/L,	158 (144–174)	158 147–178)	157 (141–173)	0.073
HCT, %	44.6 (41.0–49.0)	44.9 (41.8–49.9)	44.0 (40.1–47.7)	**0.027**
Serum ALT, U/L	24.0 (16.0–36.0)	25.0 (16.0–36.0)	23.0 (15.0–36.0)	0.400
Serum AST, U/L	29.4 (22.0–47.0)	33.0 (23.0–52.0)	27.0 (20.0–42.0)	**0.007**
Serum TB, umol/L	14.2 (9.9–20.9)	15.2 (10.7–22.9)	13.0 (9.7–19.6)	**0.015**
Serum DB, umol/L	3.4 (2.0–5.2)	3.8 (2.5–5.9)	3.0 (1.9–4.7)	**0.002**
Serum TG, mmol/L	18.1 (13.9–27.0)	18.1 (14.0–27.0)	18.0 (13.9–27.0)	0.621
Serum GLU, mmol/L	12.6 (8.5–17.4)	12.2 (8.5–16.8)	13.9 (8.5–17.6)	0.318
Serum BUN, mmol/L	4.8 (3.5–6.8)	4.9 (3.5–7.1)	4.8 (3.5–6.4)	0.521
Serum Cr, umol/L	66.0 (53.5–91.5)	68.0 (56.1–102.0)	62.7 (50.8–86.2)	**0.033**
Serum Ca, mmol/L	2.0 (1.7–2.2)	2.0 (1.7–2.2)	2.0 (1.7–2.2)	0.549
Serum CRP, mg/L	214 (134–372)	250.0 (145.0–384.0)	197.0 (121.0–334.0)	0.060

OMA, occasional or moderate alcohol; WA, without alcohol; IQR, interquartile range; CTSI, CT severity index; APACHE II, acute physiology and chronic health evaluation II; WBC, white blood count; Hb, hemoglobin; HCT, hematocrit; ALT, alanine aminotransferase; AST, aspartate transaminase; TB, total bilirubin; DB, direct bilirubin; TG, triglyceride; GLU, glucose; BUN, blood urea nitrogen; Cr, creatinine; Ca, calcium; CRP, c-reactive protein.

**Table 3:** Comparison of clinical outcomes in patients in OMA group and WA group.

Variables	Total	OMA Group (n = 188)	WA Group (n = 185)	*p*
**Primary outcomes, n (%)**				
SAP	134 (35.9)	77 (41.0)	57 (30.8)	**0.041**
Necrotizing pancreatitis	131 (35.1)	76 (40.4)	55 (29.7)	**0.030**
**Secondary outcomes**				
Persistent organ failure, n (%)	134 (35.9)	77 (41.0)	57 (30.8)	**0.041**
Persistent respiratory failure	130 (34.9)	76 (40.4)	54 (29.2)	**0.023**
Persistent renal failure	48 (12.9)	30 (16.0)	18 (9.7)	0.073
Persistent circulatory failure	25 (6.7)	16 (8.5)	9 (4.9)	0.159
Persistent MOF, n (%)	51 (13.7)	34 (18.1)	17 (9.2)	**0.012**
ACS, n (%)	23 (6.2)	13 (6.9)	10 (5.4)	0.545
Sepsis, n (%)	31 (8.3)	20 (10.6)	11 (6.0)	0.101
IPN, n (%)	42 (11.3)	25 (13.3)	17 (9.2)	0.209
Organ function support, n (%)				
CRRT	57 (15.3)	31 (16.5)	26 (14.1)	0.513
Mechanical ventilation	75 (20.1)	48 (25.5)	27 (14.6)	**0.008**
Local complications, n (%)				
APFC	133 (35.7)	61 (32.4)	72 (38.9)	0.192
ANC	131 (35.1)	76 (40.4)	55 (29.7)	**0.030**
PPC	3 (0.8)	2 (1.1)	1 (0.5)	1
WON	35 (9.4)	21 (11.2)	14 (7.6)	0.233
Interventions of local complications, n (%)	42 (11.3)	27 (14.4)	15 (8.1%)	0.056
PCD	38 (10.2)	24 (12.8)	14 (7.6)	0.097
ETD	7 (1.9)	5 (2.7)	2 (1.1)	0.449
ETN	14 (3.8)	8 (4.3)	6 (3.2)	0.607
ON	4 (1.1)	4 (2.1)	0 (0)	0.123
Length of hospital stay, days, IQR	11.0 (7.0–18.0)	12.0 (7.0–19.0)	10.0 (7.0–16.0)	0.157
Hospital total costs, yuan, IQR	26,993 (14,538–57,137)	31,118 (16,287–66,847)	23,999 (12,466–46,117)	**0.016**
ICU admission, n (%)	139 (37.3)	78 (41.5)	61 (33.0)	0.089
Dead, n (%)	26 (7.0)	16 (8.5)	10 (5.4)	0.239

OMA, occasional or moderate alcohol; WA, without alcohol; SAP, severe acute pancreatitis; MOF, multiple organ failure; ACS, abdominal compartment syndrome; IPN, infected pancreatic necrosis; CRRT, continuous renal replacement therapy; APFC, acute peripancreatic fluid collection; ANC, acute necrotic collection; WON, walled-off necrosis; PPC, pancreatic pseudocyst; PCD, percutaneous drainage; ETD, endoscopic transmural drainage; ETN, endoscopic transmural necrosectomy; ON, operative necrosectomy; ICU, intensive care unit; IQR, interquartile range.

**Table 4:** Multivariate logistic regression analysis for the association of patients’ demographic characteristics with the risk of SAP and necrotizing pancreatitis in OMA group and WA group.

	OR (95% CI)	*p*
**SAP**		
Transfer status (ref: no)		
Yes	1.40 (0.90, 2.18)	0.132
Hypertension (ref: no)		
Yes	1.52 (0.86, 2.67)	0.15
Hyperlipidemia (ref: no)		
Yes	1.32 (0.78, 2.22)	0.303
Drinking status (ref: without alcohol)		
occasional or moderate alcohol	1.57 (1.02, 2.41)	**0.041**
**Necrotizing pancreatitis**		
Age, year	1.02 (1.00, 1.04)	0.083
Transfer status (ref: no)		
Yes	1.89 (1.20, 2.97)	**0.006**
Drinking status (ref: without alcohol)		
occasional or moderate alcohol	1.60 (1.04, 2.48)	**0.034**

OMA, occasional or moderate alcohol; WA, without alcohol; SAP, severe acute pancreatitis; OR, odds ratio; CI, confidence interval. Factors associated with adverse clinical outcomes in unadjusted models (*p* < 0.2) were included in the multivariable models.

**Table 5:** Comparison of clinical outcomes in patients in DW group and WDW group.

Variables	Total	DW (n = 56)	WDW (n = 132)	*p*
**Primary outcomes, n (%)**				
SAP	77 (41.0)	32 (57.1)	45 (34.1)	**0.003**
Necrotizing pancreatitis	76 (40.4)	30 (53.6)	46 (34.8)	**<0.017**
**Secondary outcomes**				
Persistent organ failure, n (%)	77 (41.0)	32 (57.1)	45 (34.1)	**0.003**
Persistent respiratory failure	76 (40.4)	31 (55.4)	45 (34.1)	**0.007**
Persistent renal failure	30 (16.0)	13 (23.2)	17 (12.9)	0.077
Persistent circulatory failure	16 (8.5)	5 (8.9)	11 (8.3)	1
Persistent MOF, n (%)	34 (18.1)	13 (23.2)	21 (15.9)	0.234
ACS, n (%)	13 (6.9)	6 (10.7)	7 (5.3)	0.306
Sepsis, n (%)	20 (10.6)	9 (16.1)	11 (8.3)	0.116
IPN, n (%)	25 (13.3)	11 (19.6)	14 (10.6)	0.095
Organ function support, n (%)				
CRRT	31 (16.5)	12 (21.4)	19 (14.4)	0.235
Mechanical ventilation	48 (25.5)	19 (33.9)	29 (22.0)	0.085
Local complications, n (%)				
APFC	61 (32.4)	13 (23.2)	48 (36.4)	0.078
ANC	76 (40.4)	30 (53.6)	46 (34.8)	**<0.017**
PPC	2 (1.1)	1 (1.8)	1 (0.8)	0.508
WON	21 (11.2)	10 (17.9)	11 (8.3)	0.058
Interventions of local complications, n (%)	27 (14.4)	11 (19.6)	16 (12.1)	0.179
PCD	24 (12.8)	10 (17.9)	14 (10.6)	0.173
ETD	5 (2.7)	0 (0)	5 (3.8)	0.327
ETN	8 (4.3)	2 (3.6)	6 (4.5)	1
ON	4 (2.1)	2 (3.6)	2 (1.5)	0.733
Length of hospital stay, days, IQR	12.0 (7.0–19.0)	16.5 (8.0–24.0)	11.0 (7.0–16.0)	**0.019**
Hospital total costs, yuan, IQR	31,118 (16,287–66,847)	51,366 (22,230–11,2063)	26,603 (15,486–54,197)	**0.008**
ICU admission, n (%)	78 (41.5)	32 (57.1)	46 (34.8)	**0.005**
Dead, n (%)	16 (8.5)	6 (10.7)	10 (7.6)	0.675

DW, drinking within 48 h before onset; WDW, without drinking within 48 h before onset; SAP, severe acute pancreatitis; MOF, multiple organ failure; ACS, abdominal compartment syndrome; IPN, infected pancreatic necrosis; CRRT, continuous renal replacement therapy; APFC, acute peripancreatic fluid collection; ANC, acute necrotic collection; WON, walled-off necrosis; PPC, pancreatic pseudocyst; PCD, percutaneous drainage; ETD, endoscopic transmural drainage; ETN, endoscopic transmural necrosectomy; ON, operative necrosectomy; ICU, intensive care unit; IQR, interquartile range.

**Table 6:** Multivariate logistic regression analysis for the association of patients’ demographic characteristics with the risk of SAP and necrotizing pancreatitis among DW group, WDW group and WA group.

	OR (95% CI)	*p*
**SAP**		
Transfer status (ref: no)		
Yes	1.35 (0.86, 2.11)	0.187
Hypertension (ref: no)		
Yes	1.67 (0.94, 2.96)	0.081
Hyperlipidemia (ref: no)		
Yes	1.29 (0.76, 2.18)	0.353
Drinking status (ref: without alcohol)		
without drinking within 48 h before onset (WDW)	1.16 (0.72, 1.88)	0.544
drinking within 48 h before onset (DW)	3.09 (1.66, 5.77)	**<0.001**
**Necrotizing pancreatitis**		
Age, year	1.02 (1.00, 1.05)	0.06
Transfer status (ref: no)		
Yes	1.84 (1.17, 2.89)	**0.009**
Drinking status (ref: without alcohol)		
without drinking within 48 h before onset (WDW)	1.26 (0.78, 2.05)	0.345
drinking within 48 h before onset (DW)	2.71 (1.46, 5.05)	**0.002**

DW, drinking within 48 h before onset; WDW, without drinking within 48 h before onset; WA, without alcohol; SAP, severe acute pancreatitis; OR, odds ratio; CI, confidence interval. Factors associated with adverse clinical outcomes in unadjusted models (*p* < 0.2) were included in the multivariable models.

## Data Availability

The data presented in this study are available on request from the corresponding author. The data are not publicly available due to privacy.

## Supplementary Materials

The following supporting information can be downloaded at: https://www.mdpi.com/article/10.3390/jcm12072566/s1, Table S1: Definitions of primary and secondary outcomes; Table S2. Univariate logistic regression analysis for SAP in OMA group and WA group; Table S3. Univariate logistic regression analysis for necrotizing pancreatitis in OMA group and WA group; Table S4. Comparison of clinical characteristics in patients in DW group and WDW group; Table S5. Comparison of clinical scoring systems and laboratory findings in patients in DW group and WDW group; Table S6. Univariate logistic regression analysis for SAP among DW group, WDW group and WA group; Table S7. Univariate logistic regression analysis for necrotizing pancreatitis among DW group, WDW group and WA group. References [24,25] are cited in the supplementary materials.
