# Supplementary material for: Alcohol Consumption within 48 hours before Onset Is Associated with Adverse Clinical Outcomes in Hypertriglyceridemic Pancreatitis
Source: J Clin Med. 2023 Mar 29;12(7):2566. doi: 10.3390/jcm12072566 (PMC10095359; doi:10.3390/jcm12072566)
Supplement: Supplementary file 1 [file jcm-12-02566-s001.zip › jcm-2188313-supplementary.pdf]

Supplemental Tables

Table S1. Definitions of primary and secondary outcomes

**Define terms**	**Definition**	**Reference**
SAP	Presence of organ failure for more than 48 hours	[14] Gut. 2013;62(1):102-11
Necrotizing pancreatitis	A nonenhanced area of the pancreatic parenchyma, peripancreatic tissue, or both on contrast-enhanced CT	[14] Gut. 2013;62(1):102-11
Persistent organ failure	Organ failure for more than 48 hours	[14] Gut. 2013;62(1):102-11
Persistent respiratory failure	$PaO_2/FiO_2 < 300$ for more than 48 hours	[14] Gut. 2013;62(1):102-11
Persistent renal failure	Serum creatinine level > 177 µmol/L after rehydration for more than 48 hours	[14] Gut. 2013;62(1):102-11
Persistent circulatory failure	Systolic blood pressure < 90 mmHg, despite adequate fluid resuscitation	[14] Gut. 2013;62(1):102-11
Persistent MOF	Failure of two or more organs for more than 48 hours	[14] Gut. 2013;62(1):102-11
ACS	A sustained intra-abdominal pressure (IAP) > 20 mmHg that is associated with new organ dysfunction/failure	[24] Intensive Care Med. 2013;39(7):1190-206
Sepsis	The presence of infection as well as the systemic manifestations based on the surviving sepsis campaign (SSC) 2012	[25] Intensive Care Med. 2013;39(2):165-228

IPN	At least one of the following criteria was met: the presence of extraluminal gas in the pancreatic and/or peripancreatic tissues on contrast-enhanced computed tomography (CECT); a positive bacterial and/or fungi on Gram stain or culture of pancreatic and/or peripancreatic necrotic tissues or fluids obtained by percutaneous fine-needle aspiration or from drainage	[14] Gut. 2013;62(1):102-11
APFC	It was diagnosed when the following criteria on CECT were met:(a) Seen in the peripancreatic fluid areas within the first 4 weeks after the onset of interstitial oedematous pancreatitis. (b)Homogeneous collection with fluid density. (c)Confined by normal peripancreatic fascial planes. (d)No definable wall encapsulating the collection. (e)Adjacent to pancreas (no intrapancreatic extension)	[14] Gut. 2013;62(1):102-11
ANC	It was diagnosed when the following criteria on CECT were met:(a) Seen in the intrapancreatic and/or extrapancreatic collection within the first 4 weeks after the onset of acute necrotizing pancreatitis. (b)Collection containing heterogeneous and non-liquid density of varying degrees(some appear homogeneous early in their course) (c) No definable wall encapsulating the collection.	[14] Gut. 2013;62(1):102-11

PPC	It was diagnosed when the following criteria on CECT were met:(a) Maturation usually required more than 4 weeks after the onset of interstitial oedematous pancreatitis. (b)Well circumscribed. (c)Homogeneous fluid density. (d)Only liquid component. (e)Well-defined wall(completely encapsulated)	[14] Gut. 2013;62(1):102-11
WON	It was diagnosed when the following criteria on CECT were met:(a) Maturation usually required more than 4 weeks after the onset of acute necrotizing pancreatitis. (b)Seen in the intrapancreatic and/or extrapancreatic. (c) Heterogeneous with liquid and non-liquid density with varying degrees of loculations (some may appear homogeneous). (d) Well-defined wall (completely encapsulated)	[14] Gut. 2013;62(1):102-11

SAP, severe acute pancreatitis; MOF, multiple organ failure; ACS, abdominal compartment syndrome; IPN, infected pancreatic necrosis; APFC, acute peripancreatic fluid collection; ANC, acute necrotic collection; WON, walled-off necrosis

Table S2. Univariate logistic regression analysis for SAP in OMA group and WA group

	Unadjusted OR (95% CI)	P
Sex (ref: male)		
Female	0.97 (0.56, 1.68)	0.905
Age, year	1.00 (0.98, 1.02)	0.813

BMI, kg/m2	1.01 (0.95, 1.07)	0.749
Transfer status (ref: no)		
Yes	1.38 (0.90, 2.13)	0.144
History of smoking (ref: no)		
Yes	0.92 (0.59, 1.42)	0.701
Hypertension (ref: no)		
Yes	1.56 (0.90, 2.68)	0.110
Diabetes mellitus (ref: no)		
Yes	0.91 (0.54, 1.55)	0.727
Hyperlipidemia (ref: no)		
Yes	1.48 (0.90, 2.45)	0.124
COPD (ref: no)		
Yes	1.79 (0.11-28.84)	0.682
Coronary artery disease (ref: no)		
Yes	-	0.979
Drinking status (ref: without alcohol)		
occasional or moderate alcohol	1.56 (1.02, 2.39)	0.042

OMA, occasional or moderate alcohol; WA, without alcohol; SAP, severe acute pancreatitis; OR, odds ratio; CI, confidence interval; BMI, body mass index; COPD, chronic obstructive pulmonary disease; P<0.05 was bolded.

Table S3. Univariate logistic regression analysis for necrotizing pancreatitis in OMA group and WA group

	Unadjusted OR (95% CI)	P
Sex (ref: male)		
Female	0.79 (0.45, 1.39)	0.419
Age, year	1.02 (1.00, 1.04)	0.082

BMI, kg/m2	0.99 (0.93, 1.06)	0.805
Transfer status (ref: no)		
Yes	1.86 (1.19, 2.90)	0.007
History of smoking (ref: no)		
Yes	1.18 (0.77, 1.83)	0.445
Hypertension (ref: no)		
Yes	1.39 (0.81, 2.41)	0.234
Diabetes mellitus (ref: no)		
Yes	0.95 (0.56, 1.62)	0.852
Hyperlipidemia (ref: no)		
Yes	0.90 (0.54, 1.52)	0.703
COPD (ref: no)		
Yes	1.85 (0.12-29.88)	0.663
Coronary artery disease (ref: no)		
Yes	-	0.979
Drinking status (ref: without alcohol)		
occasional or moderate alcohol	1.60 (1.04, 2.46)	0.031

OMA, occasional or moderate alcohol; WA, without alcohol; OR, odds ratio; CI, confidence interval; BMI, body mass index; COPD, chronic obstructive pulmonary disease; P<0.05 was bolded.

Table S4. Comparison of clinical characteristics in patients in DW group and WDW group

**Variables**	**Total**	**DW group (n=56)**	**WDW group (n=132)**	**P**
Sex(male), n (%)	180 (95.7)	54 (96.4)	126 (95.5)	1

Age, years, IQR	42.0 (35.0-47.0)	41.0 (34.8-45.2)	42.0 (35.0-48.0)	0.280
BMI, kg/m^2, IQR	26.1 (24.2-27.8)	25.8 (24.1-26.9)	26.3 (24.2-27.9)	0.303
Transfer status, n (%)	110 (58.5)	37 (66.1)	73 (55.3)	0.171
History of smoking, n (%)	116 (61.7)	29 (51.8)	87 (65.9)	0.068
Comorbidities, n (%)				
Hypertension	31 (16.5)	5 (8.9)	26 (19.7)	0.069
Diabetes mellitus	40 (21.3)	10 (17.9)	30 (22.7)	0.456
Hyperlipidemia	44 (23.4)	14 (25.0)	30 (22.7)	0.736
COPD	1 (0.5)	0 (0)	1 (0.8)	1
Coronary artery disease	1 (0.5)	0 (0)	1 (0.8)	1

DW, drinking within 48h before onset; WDW, without drinking within 48h before onset; IQR, Interquartile Range; BMI, body mass index; COPD, chronic obstructive pulmonary disease

Table S5. Comparison of clinical scoring systems and laboratory findings in patients in DW group and WDW group

Variables	Total	DW (n=56)	WDW (n=132)	P
Clinical score systems, median (IQR)				
CTSI score ≥ 4, n (%)	82 (43.6)	26 (46.4)	56 (42.4)	0.613
Ranson score ≥ 3, n (%)	102 (54.3)	27 (48.2)	75 (56.8)	0.279
APACHE II score ≥ 8, n (%)	91 (48.4)	31 (55.4)	60 (45.5)	0.214
Laboratory findings, median (IQR)				
WBC, × 10^9/L	12.6 (10.0-16.0)	12.9 (10.2-15.2)	12.6 (10.0-16.0)	0.921
Hb, g/L,	158 147-178)	158 (148-174)	158 (147-180)	0.897
HCT, %	44.9 (41.8-49.9)	45.0 (41.1-48.8)	44.8 (42.2-50.1)	0.627
Serum ALT , U/L	25.0 (16.0-36.0)	25.0 (17.5-36.2)	25.0 (16.0-35.0)	0.722
Serum AST , U/L	33.0 (23.0-52.0)	40.0 (23.0-68.0)	30.0 (23.0-47.0)	**0.045**
Serum TB , umol/L	15.2 (10.7-22.9)	16.0 (13.0-25.9)	14.0 (9.9-22.5)	**0.048**
Serum DB , umol/L	3.8 (2.5-5.9)	4.1 (2.9-7.8)	3.7 (2.4-5.6)	0.111

Serum TG , mmol/L	18.1 (14.0-27.0)	20.0 (14.1-27.7)	17.9 (14.0-26.0)	0.584
Serum GLU , mmol/L	12.2 (8.5-16.8)	10.2 (7.6-15.9)	12.5 (9.5-17.1)	0.072
Serum BUN , mmol/L	4.9 (3.5-7.1)	5.6 (3.8-8.8)	4.6 (3.3-6.4)	0.064
Serum Cr , umol/L	68.0 (56.1-102.0)	70.3 (55.8-158.0)	67.6 (56.1-96.1)	0.662
Serum Ca, mmol/L	2.0 (1.7-2.2)	1.9 (1.5-2.2)	2.0 (1.8-2.2)	0.058
Serum CRP, mg/L	250.0 (145.0-384.0)	248.0 (136.0-368.0)	257.0 (150.0-388.0)	0.375

DW, drinking within 48h before onset; WDW, without drinking within 48h before onset; IQR, Interquartile Range; CTSI, CT severity index; APACHE II, acute physiology and chronic health evaluation II; WBC: white blood count; Hb, hemoglobin; HCT: hematocrit; ALT: alanine aminotransferase; AST: aspartate transaminase; TB: total bilirubin; DB: direct bilirubin; TG: triglyceride; GLU: glucose; BUN: blood urea nitrogen; Cr: creatinine; Ca: calcium; CRP: c-reactive protein.

Table S6. Univariate logistic regression analysis for SAP among DW group, WDW group and WA group

	Unadjusted OR (95% CI)	P
Sex (ref: male)		
Female	0.97 (0.56, 1.68)	0.905
Age, year	1.00 (0.98, 1.02)	0.813
BMI, kg/m2	1.01 (0.95, 1.07)	0.749
Transfer status (ref: no)		
Yes	1.38 (0.90, 2.13)	0.144

History of smoking (ref: no)		
Yes	0.92 (0.59, 1.42)	0.701
Hypertension (ref: no)		
Yes	1.56 (0.90, 2.68)	0.110
Diabetes mellitus (ref: no)		
Yes	0.91 (0.54, 1.55)	0.727
Hyperlipidemia (ref: no)		
Yes	1.48 (0.90, 2.45)	0.124
COPD (ref: no)		
Yes	1.79 (0.11-28.84)	0.682
Coronary artery disease (ref: no)		
Yes	-	0.979
Drinking status (ref: without alcohol)		
without drinking within 48h before onset (WDW)	1.16 (0.72, 1.87)	0.534
drinking within 48h before onset (DW)	2.99 (1.62, 5.54)	< 0.001

SAP, severe acute pancreatitis; DW, drinking within 48h before onset; WDW, without drinking within 48h before onset; WA, without alcohol; OR, odds ratio; CI, confidence interval; BMI, body mass index; COPD, chronic obstructive pulmonary disease; P<0.05 was bolded.

Table S7. Univariate logistic regression analysis for necrotizing pancreatitis among DW group, WDW group and WA group

	Unadjusted OR (95% CI)	P
Sex (ref: male)		
Female	0.79 (0.45, 1.39)	0.419
Age, year	1.02 (1.00, 1.04)	0.082
BMI, kg/m2	0.99 (0.93, 1.06)	0.805
Transfer status (ref: no)		
Yes	1.86 (1.19, 2.90)	0.007

History of smoking (ref: no)		
Yes	1.18 (0.77, 1.83)	0.445
Hypertension (ref: no)		
Yes	1.39 (0.81, 2.41)	0.234
Diabetes mellitus (ref: no)		
Yes	0.95 (0.56, 1.62)	0.852
Hyperlipidemia (ref: no)		
Yes	0.90 (0.54, 1.52)	0.703
COPD (ref: no)		
Yes	1.85 (0.12-29.88)	0.663
Coronary artery disease (ref: no)		
Yes	-	0.979
Drinking status (ref: without alcohol)		
without drinking within 48h before onset (WDW)	1.26 (0.78, 2.04)	0.335
drinking within 48h before onset (DW)	2.73 (1.48, 5.03)	**0.001**

DW, drinking within 48h before onset; WDW, without drinking within 48h before onset; WA, without alcohol; OR, odds ratio; CI, confidence interval; BMI, body mass index; COPD, chronic obstructive pulmonary disease; P<0.05 was bolded.
